# Proposal for a Radiological Classification System for Carpo-Metacarpal Joint Dislocations with or without Fractures

**DOI:** 10.5704/MOJ.1807.008

**Published:** 2018-07

**Authors:** GT Pundkare, SS Deshpande

**Affiliations:** Department of Orthopaedics, Bharati Vidyapeeth Deemed University Medical College and Hospital, Pune, India

**Keywords:** carpo-metacarpal (CMC) joint, dislocation, classification

## Abstract

**Introduction:** Though complex injuries like CarpoMetacarpal (CMC) Joint dislocations represent only 1% of all hand injuries, they have disabling impact on the functional status of patient. There are no reports in the literature classifying disabling complex injuries like CMC joint dislocations presumably because of low incidence. We propose a new classification through retrospective analysis of patients, along with literature search.

**Materials and Methods:** A new classification system has been proposed and designed at our clinical unit and applied to eight patients with CMC joint dislocations. All patients were treated with open reduction with Kirschner wire fixation. At follow-up all these patients were analysed for radiographic assessments and functional scores.

**Results:** The proposed classification identifies three types of dislocations and an additional complex category to supplement any basic type. The direction of dislocation describes the types as Type A: Dorsal, Type B: Volar and Type C: Divergent. Among the eight patients in our study, we had two of Type A, two Type B, three Type B.1, one Type C. 1. These patients had average follow-up of 18 months. The quick DASH score improved from 75.76 at 6 weeks to 1.9 at 18 months. We also did intra-observer and inter-observer reliability which scored 1.

**Conclusion:** Our proposal is a reproducible, simple, comprehensive and practical classification, easily remembered and communicated among colleagues. It is clinically relevant as it helps us in planning surgical management and prognostic evaluation.

## Introduction

Carpo-Metacarpal Joint (CMC) dislocations with or without associated fractures are not commonly encountered and represent 1% of all hand injuries^1^. Though the percentage is small these injuries are complex and have the potential for long term limitations or disabilities. Henderson stated that up to 70% injuries were missed or misdiagnosed^2^. When the CMC injuries are part of the poly-trauma injury complex the treatment is often delayed or missed in the process of attention to grave or major injuries. The delayed diagnosis is usually due to significant swelling, overlap on the oblique radiograph^3^ or inappropriate radiographs evaluation.

In spite of making the diagnosis there is still no consensus in the orthopaedic fraternity regarding approach to treatment. There have been many case reports and a few small series in the literature but no classification has been suggested, presumably because of the low incidence of this injury. Hence we felt the need for a classification to improve awareness, to identify and to facilitate clinical grouping of patients, and to provide a guideline for prognostication. The classification system proposed and developed by us is clinically relevant, based on commonly available investigation like radiographs, reproducible, easy to remember and apply in the management of these injuries. The classification, by detailing the indicator points, will particularly help in those areas in which the surgeon may fail to notice the injury.

## Materials and Methods

All consecutive patients presented with this complex hand injury during the period January 2010 to May 2016 were included in the study. The diagnosis was made with AP, oblique and true lateral radiographs of the hand. All the eight patients with CMC injuries were treated with open reduction and Kirschner wire fixation soon after diagnosis, and subjected to a retrospective analysis. They were reviewed again during follow-up and clinical functional assessment with DASH scores. Their radiographs were then studied to devise the new classification system. The printed radiograph films of all eight patients were given to five senior orthopaedic surgeons to apply our classification. This helped us to observe inter-observer reliability. The same radiographs were given to the same observers after a gap of one week to assess intra-observer reliability. After one week radiographs were given at random without the printed classification to assess “recall test” and to help us evaluate if the surgeon was able to remember the initial classification.

This classification should help for teaching purpose as well as to bring in uniformity in treating these injuries, thereby improving the quality of management. We have taken into consideration multiple factors while devising this classification, such as high energy or low energy, direct or indirect trauma, direction of the force of injury, associated fractures, basic anatomy of CMCs and management options. The follow-up results from our study and literature helped us to make prognostication of the injury. The direction of dislocation was considered to offer more valuable information about the possible mechanism and appropriate surgical approach. Hence, it was used as a basic principle in the classification. This single finding was very easy to establish on true lateral radiographic view of the hand. Once this first step was done attention was given to associated injuries. Greater emphasis was given to mechanism of injury and direction of dislocation rather than to the number of metacarpals involved.

**Fig. 1:**
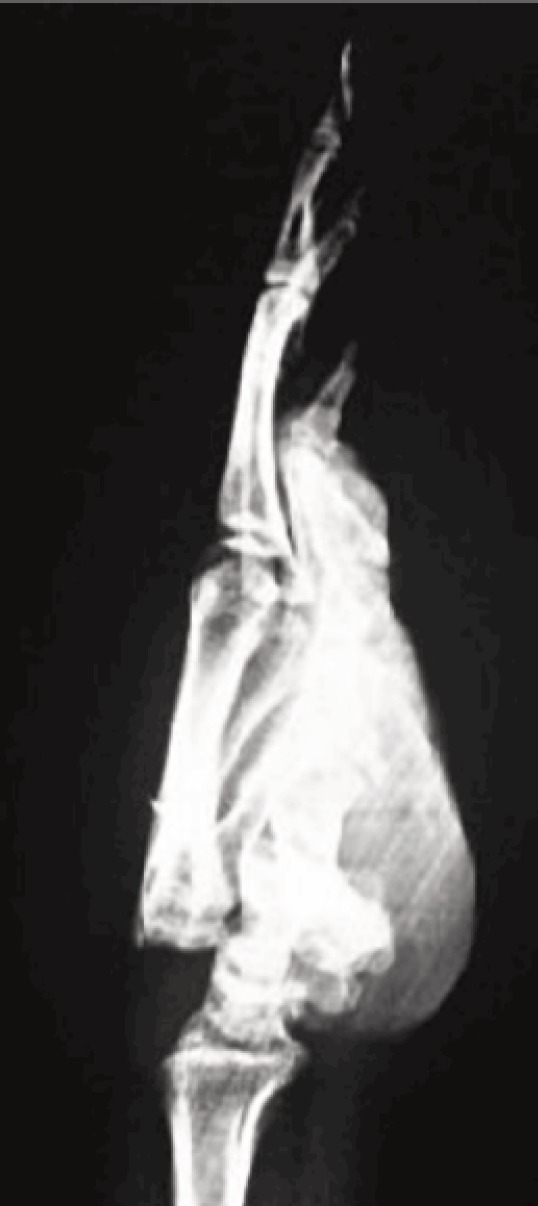
Lateral radiograph view showing Classification Type A. Dorsal dislocation of multiple metacarpals without any associated fractures.

**Fig. 2:**
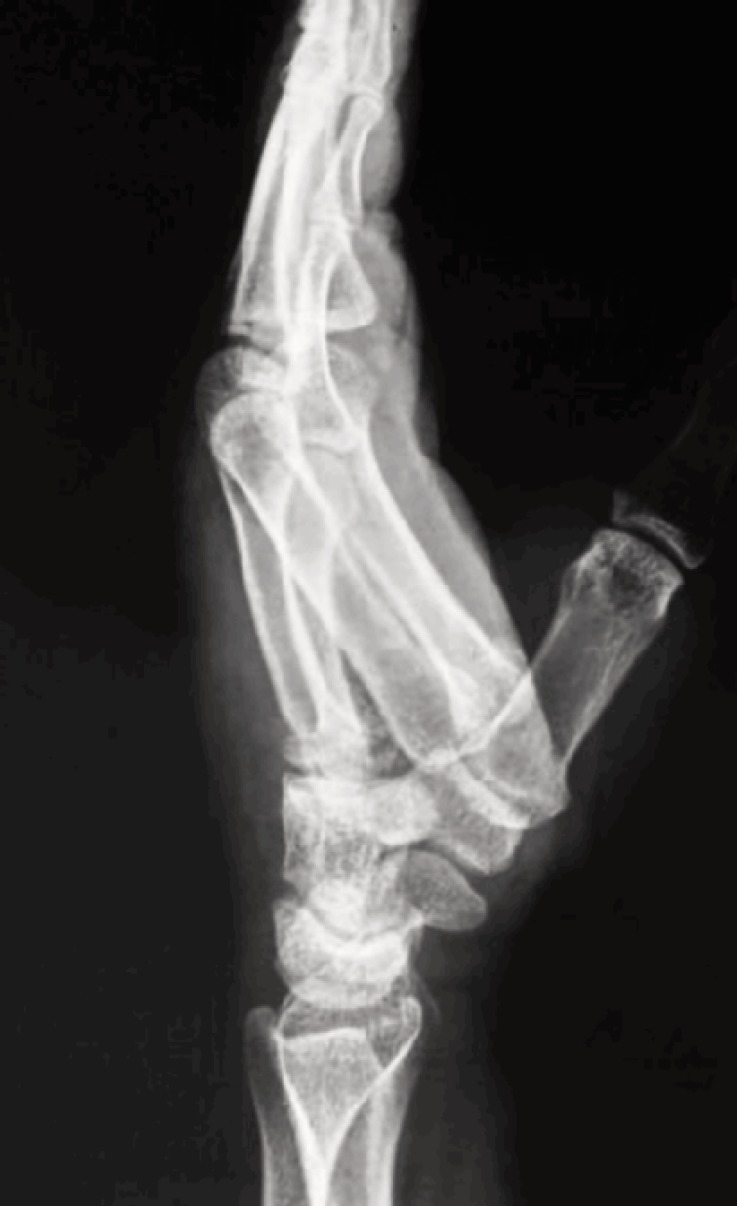
Lateral radiograph showing Classification Type B. Volar dislocation of index and middle finger metacarpals without any associated fractures.

The treatment approach remained the same for single or multiple metacarpal dislocations. However, the management in multiple dislocations in opposing directions would change and hence these injuries were given separate consideration as Type C. Along with this, the mark, decimal point 1 (.1) was added to the basic type to recognise the presence of associated fractures in carpal or metacarpal bones. This was done to focus on the need for precise intra-articular reduction for the more complex pattern of injury and high energy trauma. The proposed classification is simple to understand. One has to look at the direction of dislocation of metacarpals in the lateral view of radiographs. Three types are proposed: Type A - the direction of dislocation is dorsal in this type which easy to understand (Fig. 1); Type B is with volar dislocation (Fig. 2); Type C is unique resulting in a few metacarpals dislocated in dorsal direction and few others in volar direction.

A “divergent type” means that the metacarpals have dislocated in both dorsal and volar directions, creating a divergent ‘inverted V’ on the lateral view but the number and pattern can vary depending on the forces acting and on the mechanism of injury. To add information about complexities, like fractures in phalanges, carpals or dislocations, the numeral (.1) is added as an additional category. This emphasises and brings attention of the surgeon to associated injuries while planning and performing the surgery. So if the patient has sustained dorsal dislocation with concomitant carpal or metacarpal fracture one would classify it as Type A.1.

**Fig. 3:**
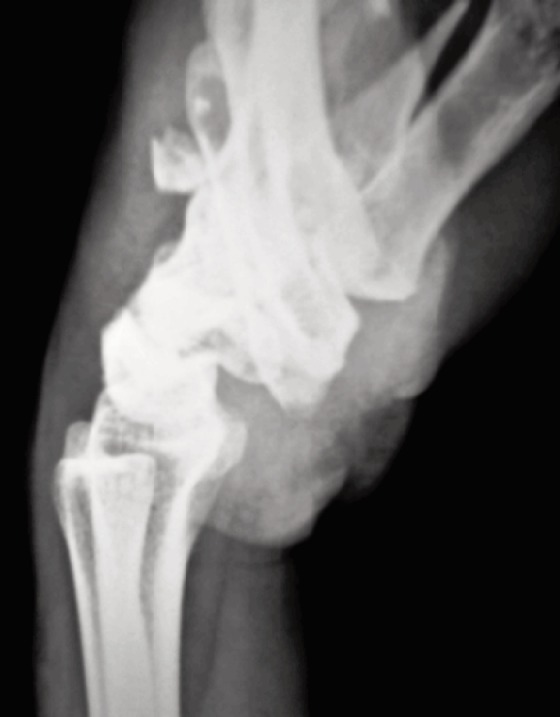
Lateral radiograph view showing Classification Type B.1. Volar dislocation of multiple metacarpals along with concomitant fracture at the base of ring metacarpal shaft.

**Fig. 4:**
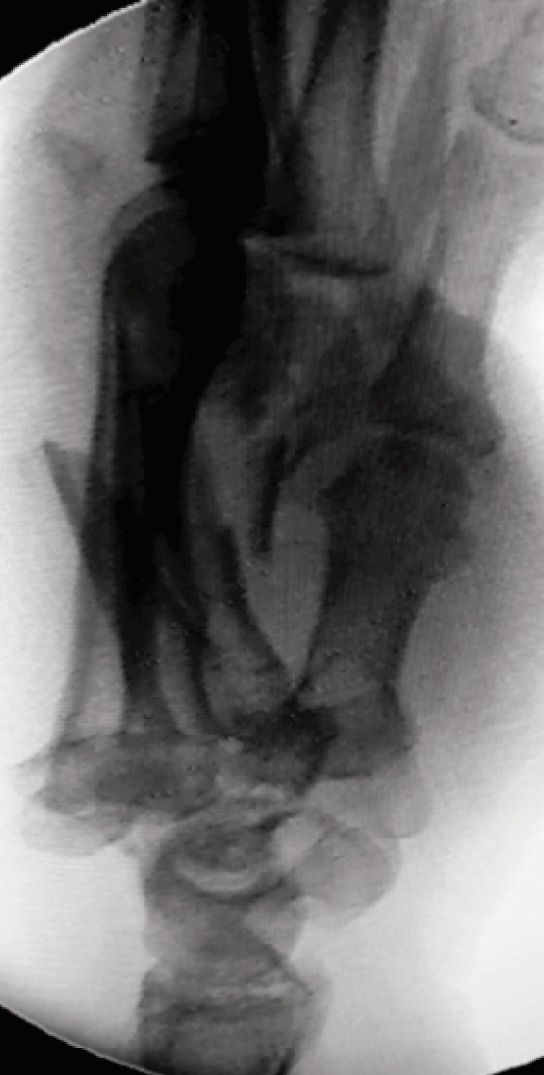
Lateral radiograph view showing Classification Type C.1. Divergent dislocations of multiple metacarpals with concomitant metacarpal shaft fractures.

The patients with volar dislocation and concomitant carpal or metacarpal fractures would be indicated as Type B.1 (Fig. 3). The divergent dislocation with concomitant carpal or metacarpal fracture would be classified as Type C.1 (Fig. 4). The types are prognosticated depending on personal experience of the surgeon and literature review (Table I). All the patients were treated with manipulation under anaesthesia with closed and/or open reduction with Kirschner wire fixation.

**Table I:** Classification of Carpo-Metacarpal (CMC) joint dislocation

Type	Direction of dislocation	Involved metacarpals	Peculiarity
A	Dorsal	1 or more	All in same direction
B	Volar	1 or more	All in same direction
C	Divergent: Dorsal and Volar	2 or more	At least two in opposite direction
To add .1	Any of the above plus concomitant carpal or metacarpal fracture	Metacarpal dislocation with associated fractures	Displaced fracture

## Results

In our series of eight patients there were two Type A, two Type B, three Type B.1, one Type C.1. These patients had average follow-up of 18 months. The quick DASH score improved from 75.76 at six weeks to 1.9 at 18 months. The divergence group had also improved in the DASH score but had prolonged recovery. The prognosis depended on early diagnosis, treatment and the type of the fracture. Radiographs of our patients could be easily classified by the system (Table II).

**Table II:** Application of proposed classification in patients in our study

Serial number	Age/Sex	Type of dislocation	Associated fractures	Our classification Type
1	22/M	Volar	Nil	B
2	56/M	Volar	Fracture shaft first metacarpal	B.1
3	19/M	Dorsal	Nil	A
4	25/M	Volar	Fracture base to shaft second metacarpal	B.1
5	24/M	Divergent	Fracture neck of fourth metacarpal	C.1
6	38/M	Volar	Nil	B
7	36/M	Dorsal	Nil	A
8	14/M	Volar	Fracture of proximal phalanx index finger	B.1

Our recall test showed that surgeons could easily classify the fractures by observing the direction of displacement. The same radiographs were given to the same observers after a lapse of one week to assess intra-observer reliability. After one week, random radiographs were given to the surgeons, showing CMC dislocations without the printed classification for reference and the surgeons could easily replicate the results. This helped to confirm that our classification system was simple, easy to apply and recall.

## Discussion

The classification systems are devised with the general aim of simplifying the diagnostic features of these complex injuries. It helps us in deciding the treatment algorithm. The use of the system takes into consideration any minor injuries which may have an impact on the prognosis. Improved peer to peer communication, clarity on severity of grade of injury and planning surgical treatment are the benefits of our classification system.

More elaborate and complicated classifications often tend to confuse the reader. While keeping it simple, at the same time the classification should give pointers towards management and prognosis. It was felt that CMC injuries are missed or diagnosed late because of lack of awareness. By devising this classification we are focusing attention on these complex injuries. To maintain simplicity the use of CT scan is not advised but good quality radiographs are adequate, with emphasis on true lateral radiograph.

Delayed diagnosis and treatment will result in degenerative arthritis and reduced grip strength^4^ and residual pain, disability, functional loss and eventual economic loss. Appropriate management can help as much as 87% patients in return to full work and sporting activity as per Lawlis^5^. Good history will be of value in the patients presenting with hand injury. Good quality AP, true lateral and 45 degrees oblique radiographs^6,7^ should be taken. Woon *et al* have explained in detail the anatomical reasons for dislocations and the direct and indirect forces acting at the time of injury. They have raised the need for high index of suspicion based of the mechanism of injury and clinical examination which will lead to early diagnosis^8^. Various mechanisms of injury have been proposed (Table III). The functional results will depend on the accurate anatomic reduction of the joints. We have reemphasised this in our previous report^9^ about the fixation of third metacarpal first for the “Key-Stone” effect which in turn helped in the reduction in multiple dislocations^10^.

**Table III:** Proposed category of mechanisms of CMC injury

**Type**	**Direction of dislocation**	**Involved Metacarpals**	**Peculiarity**	**Possible Mechanisms of Injury**
A	Dorsal	1 or more	All in same direction	A direct force applied on the palmer aspect of the metacarpal bases will cause dorsal dislocation which is in the direction of the force
B	Volar	1 or more	All in same direction	A direct force applied on the dorsal aspect of the metacarpal bases will push the metacarpals to the volar side, which is in the direction of the force causing volar dislocation
C	Divergent: Dorsal and Volar	2 or more	At least two in opposite direction	The mechanism here is quite complex involving twisting and rotatory forces. This may cause crushing or supination of the transverse metacarpal arch around an axis passing between third and fourth metacarpals

A simple classification is proposed depending on the direction of dislocation, which is easily established radiologically in spite of multiple metacarpal involvements or swelling of the hand. The severity grade and prognostication based on the grading classification is also discussed. The grading system also gives an indication of the intra-operative approach which is the same as the classification grade to correct the dislocation. Additional decimal point one (.1) category can be added to any grade of classification if radiographs show concomitant carpal or metacarpal injuries. These additional injuries include fractures of metacarpal shaft, metacarpal neck, injury to hamate, capitate^11^ or other carpal bones, and avulsion fractures^2,5,8^.

In our study, one patient had second metacarpal dorsal dislocation with injury to hamate which would be classified as A.1 type. Divergent dislocation (Type C) is the most severe form of injury. Type C mechanism is described by Dillon *et al* which are due to direct as well as indirect forces. Such complex forces often are associated carpal injuries^9^. These patients are likely to have poor results if not treated early and appropriately. The patient with divergent type injury also had significant improvement in DASH score with a prolonged recovery. The prognosis depends on the early diagnosis, treatment and the type of the fracture. When the injuries are appropriately treated, the functional results range from excellent to good. Poor results are expected in missed and late presentation cases.

The Recall Test was done among five orthopaedic surgeons. All of them were able to recall the classification and graded them easily. The intra-observer and inter-observer reliability tests were done, all of which achieved the score of “1”. The score ‘1’ represents that each surgeon was able to memorise and recall the type of classification after evaluating radiographs. This good score basically reemphasises the simplicity of the classification system avoiding errors in judgment. The direction of dislocation can be easily assessed with good study of the radiograph. This classification also gives pointers towards management of the patient and the prognosis.

## Conclusion

To our knowledge there is no classification available for CMC injuries. Our proposed system is comprehensive and adds value to the management.
